# Machine learning: assessing neurovascular signals in the prefrontal cortex with non-invasive bimodal electro-optical neuroimaging in opiate addiction

**DOI:** 10.1038/s41598-019-54316-6

**Published:** 2019-12-04

**Authors:** Hada Fong-ha Ieong, Fu Gao, Zhen Yuan

**Affiliations:** 1 Bioimging Core, Faculty of Health Sciences, University of Macau, Taipa, Macau SAR, China; 2 Department of Cardiac Surgery, Yale School of Medicine, Yale University, New Haven, CT U.S.A.; 3 Centre for Cognitive and Brain Sciences, Institute of Collaborative Innovation, University of Macau, Taipa, Macau SAR, China; 4 Present Address: Department of Anesthesiology, Yale School of Medicine, Yale University, New Haven, CT U.S.A.

**Keywords:** Predictive markers, Addiction

## Abstract

Chronic and recurrent opiate use injuries brain tissue and cause serious pathophysiological changes in hemodynamic and subsequent inflammatory responses. Prefrontal cortex (PFC) has been implicated in drug addiction. However, the mechanism underlying systems-level neuroadaptations in PFC during abstinence has not been fully characterized. The objective of our study was to determine what neural oscillatory activity contributes to the chronic effect of opiate exposure and whether the activity could be coupled to neurovascular information in the PFC. We employed resting-state functional connectivity to explore alterations in 8 patients with heroin dependency who stayed abstinent (>3 months; HD) compared with 11 control subjects. A non-invasive neuroimaging strategy was applied to combine electrophysiological signals through electroencephalography (EEG) with hemodynamic signals through functional near-infrared spectroscopy (fNIRS). The electrophysiological signals indicate neural synchrony and the oscillatory activity, and the hemodynamic signals indicate blood oxygenation in small vessels in the PFC. A supervised machine learning method was used to obtain associations between EEG and fNIRS modalities to improve precision and localization. HD patients demonstrated desynchronized lower alpha rhythms and decreased connectivity in PFC networks. Asymmetric excitability and cerebrovascular injury were also observed. This pilot study suggests that cerebrovascular injury in PFC may result from chronic opiate intake.

## Introduction

Opiate addiction, the most severe and chronic stage of opioid use disorder (OUD), is characterized as a chronically behavioral relapsing, and currently being proposed, a subsequently cerebrovascular^1–3^ disorder. Here, the use of opiates, such as heroin, morphine, or opium, leads to a clinically significant impairment in controlling intake^4^ and maintaining glutamate homeostasis^5^. Although there are existing theories^6,7^ to explain the underpinnings of the transition from cessation to relapse, it is believed that the neuropharmacological consequences of allostatic neuronal or hormonal adaptations result in craving, compulsive drug use, and other disadvantageous behaviors associated with addiction^8^. Prefrontal cortex (PFC), known for its involvement in regulating limbic reward regions and higher-order executive function, is an important target for drug addiction research, in large part due to its strong link to compulsive drug use^9^ and the negative-affective states associated with protracted abstinence^8^. Convincing evidence has showed reduced blood flow and thus deactivation in the PFC of drug addicts^9^. Hemodynamic changes and its subsequent inflammatory responses in the brain tissue injured by chronic and recurrent drug insult may comprise essential pathophysiological processes resulting in cerebral ischemia. Indeed, the neurovascular mechanisms underlying the prefrontal excitatory and inhibitory neuroadaptations during abstinence were not characterized across drug classes. Despite diverse treatment options for OUD, including medication (e.g. buprenorphine, methadone, naloxone/naltrexone)^4^, inpatient treatment, outpatient harm-reduction program, and counseling that offers cognitive-behavioral therapy, motivational enhancement therapy, or 12-step therapy, the relapse rate is extremely high (91%)^10^. Furthermore, there is no biomarker to predict relapse. At the microscopic scale, brain electrical activity greatly relies on neurovascular interactions between neurons, astrocytes and blood vessels. At the macroscopic scale, brain state-dependent network could reflect behaviors and cognition. Hence, one strategy for achieving potential markers that can possibly monitor a patient’s PFC network activity is the use of resting-state functional electro-vascular neuroimaging at the mesoscopic scale (i.e., intermediate level that explores a large population of cells and vessels).

Resting-state functional connectivity (rsFC) has received substantial attention clinically^11^, in large part because of its raising promise linking canonical microcircuit consisting excitatory glutamatergic projection neurons and inhibitory GABAergic interneurons^12,13^, genetics^14,15^ and predicting behavioral achievement and subsequent activation of complementary brain areas when performing task in humans^16–19^. Thus, rsFC is thought to serve as a systems-level marker to provide indicative information regarding to the diagnoses for different neuropsychiatric disorders^11^. Characterizing a mesoscopic-scale PFC network in opiate addiction during protracted abstinence through rsFC may provide insights for deeper understanding of the neurophysiological aspect of brain activity during recovery. Furthermore, if improved neuroimaging strategies can be developed and implemented in selective categories of individuals with OUD to prevent relapse, this may ultimately lead to long-term recovery.

Here, we report the results of a pilot study of rsFC analysis in the PFC of abstinent heroin-using patients who had a life-time history of opiate use. The objective of our study was to determine what the neural oscillatory activity contributes to the profound effect of heroin exposure on abstinence and whether these oscillations are associated with blood oxygenation. We used a bimodal non-invasive neuroimaging strategy that combined electroencephalography (EEG) to provide information about neural synchrony and oscillatory activity with functional near-infrared spectroscopy (fNIRS) to provide complementary information about the cerebral blood oxygenation in small vessels in the PFC. This bimodality has several features designed to optimize homogeneity. First, synchronization likelihood (SL) was used to capture the spatio-temporal neural synchrony and the oscillatory activity interactions among whole-brain electrodes to characterize interdependencies between times series as measured by EEG. Second, concentration changes in two chromophores of oxygenated hemoglobin (HbO) and deoxygenated hemoglobin (Hb) in PFC as measured by fNIRS were obtained to examine regional cerebral blood oxygenation (rCBO) and map functional brain networks. A regional increase in HbO concentration and a decrease in Hb concentration indicate a typical rCBO. Neuroimaging studies provide compelling intravascular evidence that regional brain activity induces a regional arteriolar vasodilation and consequently an increase in regional cerebral blood volume and cerebral blood flow (CBF)– known as the neurovascular coupling mechanism^20^ (for a recent review see^21^). At the capillary level, the increase in CBF and oxygen delivery exceeds the increase in local oxygen consumption, and therefore, CBO rises locally^22^, indirectly indicating brain activation. Third, a supervised machine learning method was used to obtain correlations between EEG and fNIRS modalities by extracting plausible components in order to provide a relatively precise detection and localization between the electrophysiological and hemodynamic signals. Finally, we limited enrollment of heroin-dependent patients at their protracted abstinence stage in the addiction cycle because their findings may more likely demonstrate a pronounced effect of heroin and, perhaps, a sign of recovery.

## Results

### Study participants

**Figure 1:**
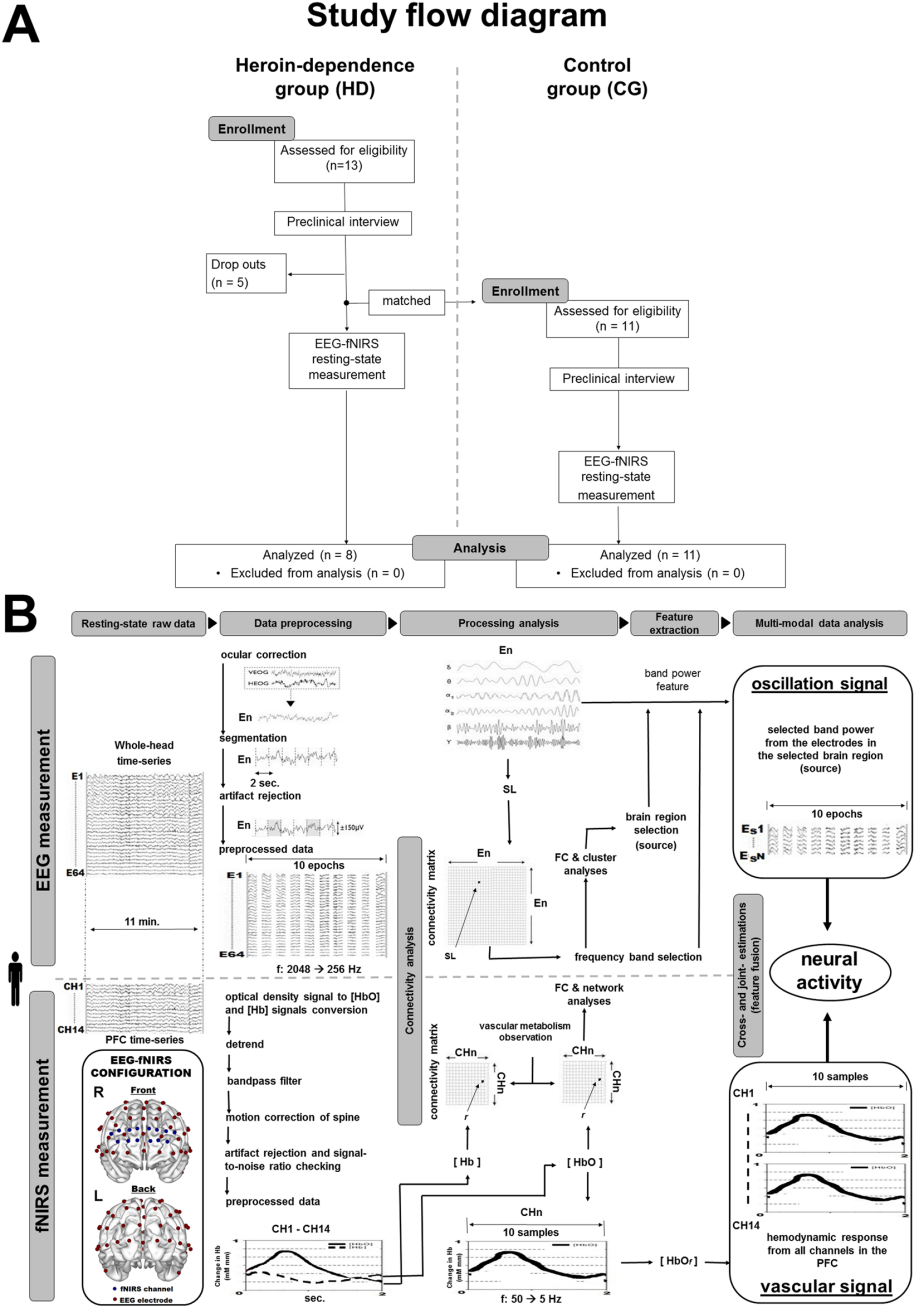
Study design. (**A**) Flow diagram for the study. (**B**) Workflow of study design. Details in Materials and Methods.

A total of 13 heroin-dependent patients (HD) who had been exposed to heroin for over 20 years and managed to stay abstinent for at least three months were enrolled from an in-patient drug rehabilitation treatment center in Macao SAR. Patients were enrolled over the course of two years. Five patients withdrew from the study before completion and were not included in the analysis. Eight patients completed the study (Fig. 1A). A total of 11 healthy subjects were enrolled from the local community as a control group (CG). The CG matched the recruited HD patients on age, education level, and IQ (Table 1).

**Table 1:** Baseline characteristics of participants.

Variable	Heroin-dependent Group (HD) (n = 8)		Control Group (CG) (n = 11)		*p*
	mean	s.d.	mean	s.d.	
Age (years)	47.6	6.1	45.2	5.52	0.40
Education (years)	5.9	2.8	7.45	2.57	0.24
IQ	82.9	11.4	80.0	10.3	0.58
Gender (M:F)	5:3		7:4		—
Duration of heroin use (years)	29.0	9.7	N/A	N/A	N/A
Duration of heroin abstinence (years)	2.39	3.4	N/A	N/A	N/A
Average heroin dosage (g/day)	0.6	0.4	N/A	N/A	N/A
Methadone treatment dosage (mg)	30.0	26.9	N/ A	N/A	N/A

The *p* values are reported for a two-sample t test (for age, years of education and IQ; two-tailed) comparing abstinent heroin-dependent subjects (HDs) with the controls (CGs). IQ was assessed with the Wechsler Adult Intelligence Scale version 4. Abbreviation: Standard deviation (s.d.).

In the day following enrollment, simultaneous whole-head EEG and PFC-localized fNIRS recordings were administered for 11 minutes when subjects were at rest, with eye-closed, and alert (Fig. 1B and Materials and Methods). No subjects discontinued participation during the resting-state recordings due to discomfort. No EEG electrodes or fNIRS channels were excluded due to poor contact on the scalp.

### Resting-state desynchronization in lower alpha rhythm in frontal and occipitoparietal cortices

**Figure 2:**
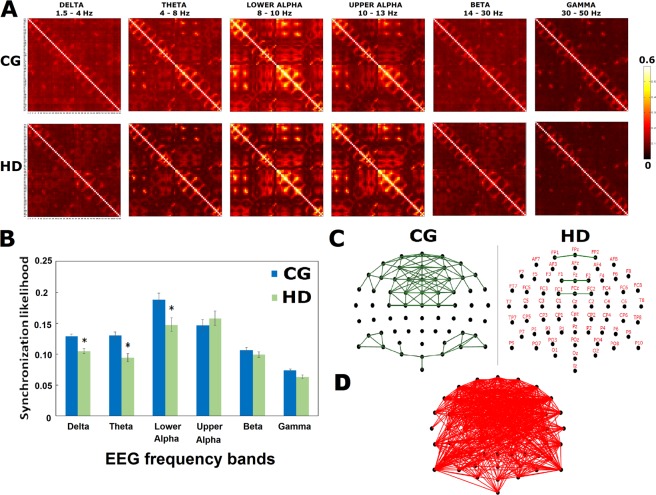
SL as a global and local connectivity by EEG. (**A**) The SL matrices, using grand-averaged values across the HD patients and the control participants are showed for each frequency band. Alpha bands are classified into two: lower alpha (8–10 Hz) and upper alpha (10–13 Hz). The number of EEG electrodes is 64, resulting in the 64 × 64 square matrix whose elements serve the average strength of SL values across the whole subjects between a pair of EEG electrodes. (**B**) Global functional connectivity indexed by mean SLs of HD patients was reduced (*p* = 0.015, permutation test) in delta-, theta-, and lower-alpha-bands. (**C**) 2D visualization of the connectivity in lower alpha based on the synchronization for a 64 EEG electrode cap. The dot (black) represents a node (i.e. EEG electrode). The segment (green) represents an edge (i.e. connectivity when the threshold is above 0.8). (**D**) Clustered connections from Cluster-based Permutation Tests (CBPT) between groups for the SL index of lower alpha band as a local connectivity. The nodes consisted of the PFC and occipitoparietal cortex comprised reduced synchronization in lower-alpha band of HD patients (*p* < 0.05, uncorrected for multiplicity).

In neurophysiological data, SL is arguably the most popularly used index to estimate generalized synchronization, which is a concept referring to a situation where the states of a dynamical (sub)system are a function of those of another (sub)system. Hence, SL measures the extent to which each pair of electrodes share mutual information that is frequency independent and it can detect linear and nonlinear dynamical interdependencies between two signals^23^ as measured by EEG. The result of computing SL for each electrode of a particular frequency band was a 64 × 64 matrix. Figure 2A shows the SL matrices for the frequency bands, classified into delta, theta, lower alpha, upper alpha, beta, and gamma, across HD patients and controls. The mean SL in the HD group decreased (*p* < 0.009) in the low frequencies, including delta, theta, and lower alpha (Fig. 2B). No between-group differences were identified for upper alpha, beta, and gamma frequency bands (all *p* > 0.19).

HD patients show desynchronization in the lower alpha frequency band in the frontal, occipital, and in part, parietal systems (Fig. 2C,D). Cluster-based Permutation Tests (CBPT) showed that the statistical effect found in comparing HD and CG groups was greater than the effects found when SL values were randomly assigned to both groups, indicating that the statistical effect was significant, and that the SL connectivity measure was sensitive to lower-alpha frequency content. Because the results were consistent with previous literature in which disturbed PFC was found in substance use disorders (SUDs) subjects^9^, the lower alpha oscillations in the PFC as a brain region were then selected as a feature for further multimodal fusion analysis (Fig. 1B).

### Decreased HbO-based resting-state functional connectivity and degree strength in PFC network

**Figure 3:**
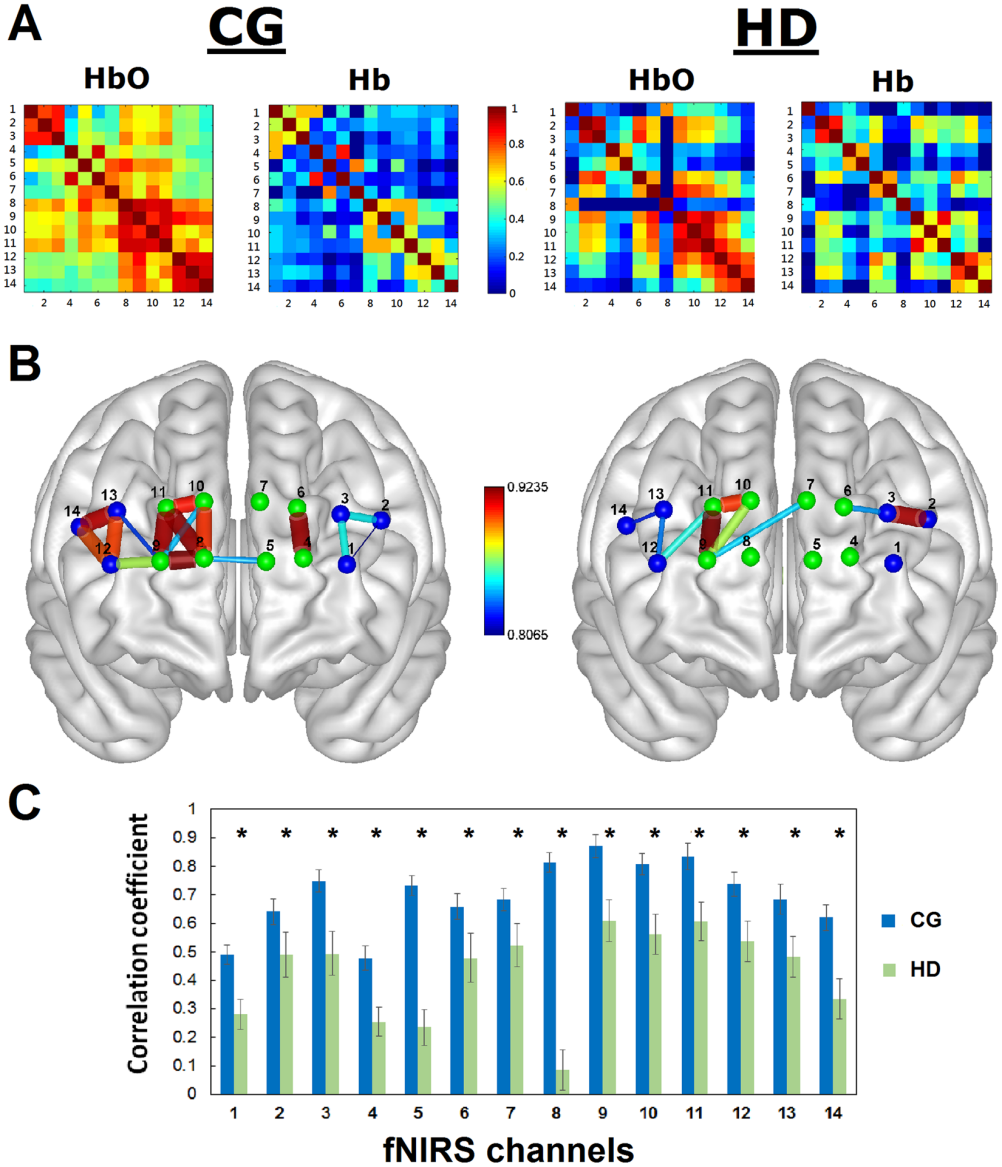
Blood oxygenation and metabolism as PFC connectivity by fNIRS. (**A**) Connectivity matrices of the HD patients and the control participants. The number of fNIRS channels is 14, resulting in the 14 × 14 square matrix whose elements represent the average correlation coefficients across the whole subjects between a pair of fNIRS channels. (**B**) Brain network patterns of the HDs and CGs. Only the topmost 5% with correlation values greater than 0.78 are shown in the figures. The nodes (green: FP; blue, dlPFC; channels) are numbered by channel, and the weighted edges (gray) are displayed. Color bars indicate the correlation coefficient values. (**C**) Degree strength with the group average of the correlation coefficients in PFC of the HD patients (green) and control subjects (blue). Error bars indicate the standard error of the mean across the HDs and CGs. Compared with the controls, the HD patients show significantly greater connectivity strength in all the channels covering the PFC in terms of HbO.

To explore the vascular response evoked by brain activity and construct the topological resting-state PFC network (Fig. 1B), changes in hemodynamic signals obtained and calculated from the 14 fNIRS channels covering the PFC were measured to construct the individual functional connectivity. Pearson correlation coefficients, *r*, among all channels were calculated by averaging the correlation matrices for each group across subjects. Figure 3A shows the group-level connectivity maps obtained from the concentration changes in HbO (see Supplementary Table S2) and Hb (see Supplementary Table S3) in the PFC of HD patients and controls. Blood metabolism in the PFC in HD patients shows less activity compared to that of controls (see Supplementary Fig. S1).

To examine the systems-level PFC network organization of the controls in comparison to the HD patients, the foremost 5% of HbO connections^24^ (correlation coefficient threshold, *r*_*T*_, >0.78; mean = 0.55) calculated in the control group were used to generate network topological properties. The organization network of HD patients demonstrated overall reduced connections in PFC topography (Fig. 3B) where nodes represent the channels and edges represent connectivity between nodes. The connectivity strength, σ, which is the average connectivity of a node in each network in the PFC system, was then quantified. Group-level analysis showed that the degree strengths were remarkably (*p* < 0.0001) lower in the HD patients compared to the controls (Fig. 3C). For simplicity, the change in HbO concentration was then selected as a feature for further multimodal fusion analysis (Fig. 1B).

### Strong correlation between lower alpha oscillations and blood oxygenation across PFC

**Figure 4:**
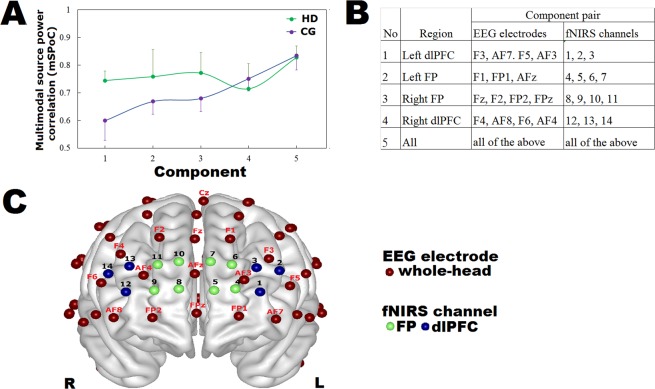
Correlation between feature extracted from EEG electrodes and fNIRS channels covered the PFC by multimodal source power comodulation analysis (mSPoC). (**A**) Correlations of lower alpha rhythm and HbO of resting-state features obtained by mSPoC. (**B**) Component pairs of EEG electrodes and their corresponding fNIRS channels. (**C**) Configuration of EEG electrodes and fNIRS channels.

The effort of combining neurophysiological signals from EEG and fNIRS modalities is known as multimodal fusion. Multimodal source power comodulation (mSPoC) analysis^25^ is a supervised machine learning method^26^ that employs the source power comodulation function^27^ for multiple classes and originates from the common spatial pattern algorithm^28^. This analysis was performed as a machine learning feature fusion method to examine whether the spectral power dynamics of the projected lower alpha signal (i.e., the EEG source) maximally covaries with the time-course of the projected HbO signal (i.e., the corresponding fNIRS source) in the PFC. The corresponding mSPoC correlations were computed, and the coupling coefficients are showed in Fig. 4A. Four regions of interest (ROIs) in the PFC were chosen (Fig. 4B) based on Montreal Neurological Institute (MNI) coordinates (see Supplementary Table S1) of the electrodes and channels (Fig. 4C). For example, electrodes F3, AF7, F5, and AF3 and channels 1, 2, and 3 corresponding to the left dorsolateral PFC (dlPFC) subregion were selected to perform multimodal fusion. To evaluate the fusion performance, the datasets extracted from the electrodes and channels covering each of the ROIs, in addition to those covering the entire PFC, were fused separately. The multimodal fusion of all the electrodes and channels covering the PFC, regardless of the ROIs, was outperformed in both HD and control groups (the strongest correction; *r*_*HD*_ = 0.83, s.e.m. = 0.04; *r*_*CG*_ = 0.83, s.e.m. = 0.05). Both HD and CG datasets show a strong relationship between lower alpha oscillations and change in HbO concentration across PFC, and thus visualization was performed later.

### Asymmetric interhemispheric excitability evidenced by hemodynamic patterns in PFC

**Figure 5:**
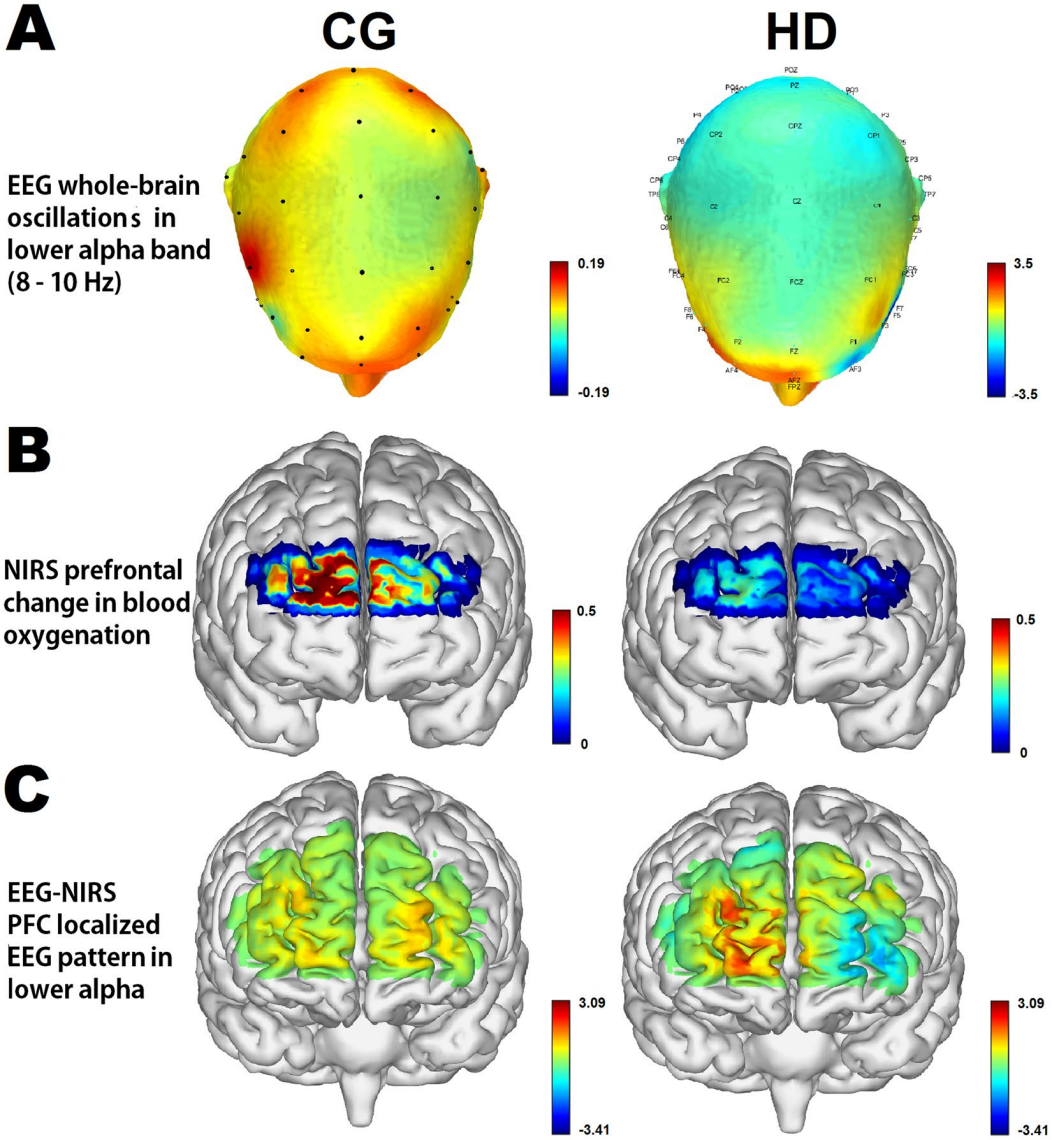
Resting-state activation pattern of the coupled EEG and fNIRS component. (**A**) Whole-brain sensor-space activation pattern of the EEG mSPoC component in lower alpha oscillations between groups. Color bar indicates the amplitude (i.e., band power) of the lower alpha frequency band in µV. (**B**) HbO-based fNIRS activation pattern of the corresponding mSPoC component in the PFC. These components were identified based on resting-state comodulation of amplitude dynamics in the EEG and the time course of HbO dynamics in the fNIRS. Color bar indicates the change in concentration of HbO (i.e., [HbO_f_]) in µM. (**C**) Estimate of the source-space pattern of the EEG component based on the sensor-space pattern after EEG-fNIRS fusion. Color bar indicates the optimized feature value of estimated components from the extract EEG and fNIRS source data.

The neurophysiological importance of alpha oscillations stems from their effect on neural excitability^29^. Figure 5 displays the activation patterns of the estimated components coupled with the EEG and fNIRS modalities by mSPoC. EEG data were bandpass-filtered to amplify oscillations in the lower alpha frequency band. HD patients show the greatest sensor-space activation pattern of lower alpha mSPoC component in the right frontopolar (FP) of PFC (AF4 electrode, MNI: x = 31, y = 61, z = 26; AFz electrode, MNI: x = 2, y = 64, z = 27; Fig. 5A) and deactivation in the left dlPFC (AF3 electrode; MNI: x = −26, y = 64, z = 23); whereas the controls show the greatest in the PFC, the occipitoparietal cortex, and in part, the right dlPFC (FC4 electrode, MNI: x = 50, y = 13, z = 51). The 2-second lower alpha oscillation dynamics from an HD patient and a control subject are provided in Supplementary Video 1. Optical density data were converted to amplify vascular responses in change in HbO concentration (i.e., blood oxygenation). Similar to the results in nodal network analysis, HD patients demonstrate decreased activation pattern of the corresponding mSPoC component in PFC (Fig. 5B).

Lower alpha band power modulations of EEG were fused with the change in HbO concentration simultaneously measured by fNIRS. Figure 5C shows the visualization of the estimate of the source-space pattern of the EEG component based on the sensor-space pattern. HD patients show interhemispheric asymmetry in PFC-localized EEG pattern in lower alpha: activation in the right FP of PFC and deactivation in the brain region between the left FP and dlPFC.

## Discussion

Functional neuroimaging has provided important contributions regarding the neuroplastic adaptations that result from chronic drug use. There are also growing separate bodies of resting-state studies demonstrating that both electrophysiology and hemodynamics can further enhance our knowledge of cognitive processing^30,31^ and improve clinical diagnostics^32–35^. Despite their raising promises to identify and predict various neuropsychiatric disease trajectories, opiate addiction has been understudied by rsFC literature. Furthermore, the fundamental mechanism that underlies the electro-vascular coupling on systems-level adaptation to chronic opiates during protracted withdrawal, which takes from months to years, are poorly understood. We found that chronic heroin-using patients after prolonged withdrawal showed desynchronization in the lower alpha frequency band, abnormal blood metabolism and decreased connectivity strength in the PFC. Enhanced electro-vascular response was found in the right PFC but reduced in the left, supporting the notion that neural oscillatory activity contributes to the chronic effect of heroin and is associated with prefrontal discontinuous neurovascular integrity. Although it is challenging to draw conclusions that the desynchronized lower rhythms and the decreased connectivity in PFC network is the result of a pathological cause (i.e., chronic heroin use) or of a therapeutic mean (i.e., remaining abstinent) from this pilot study, these findings have generated hypotheses that can be tested through future functional work in addiction models or with large clinical cohorts.

We hypothesize that the functional topological architecture of resting-state brain networks is profoundly disrupted in HD patients even after prolonged cessation from heroin. We believe this occurs especially in the lower alpha oscillations as well as in blood oxygenation variations, resulting from changes in blood flow or metabolic rate, in the PFC. This may suggest that life-time opiate addiction, in addition to the existing clinical definition, is also a cerebrovascular disorder^1–3^ that is linked to disturbed glutamate homeostasis^5^. In this study, the decreased prefrontal rCBO, and thus CBF, was associated with enhanced lower alpha oscillations in HD patients.

Neurovascular abnormality is induced by neurochemical imbalance (i.e., glutamate/GABA) in the cerebral cortex in brain disorders^36^. At the macroscopic scale, the human brain, in general, and the cortex, in particular, are organized in a hierarchical manner by brain cell assembly to constitute complex networks consisting of brain regions that manifest functional connectivity, and thus enabling cognition^16,18^, perception^37^, and behavior^19^. The fundamental processing unit throughout the cortex is the canonical microcircuit consisting of glutamatergic (i.e., excitatory) projection neurons, that support 85% of energy consumed by the brain^38^, and GABAergic (i.e., inhibitory) interneurons under functional modulation by projections to thalamus, basal forebrain and brainstem^13,39,40^. At the microscopic scale, a neurovascular unit is made of the neuron, the astrocyte glia, and the vascular smooth muscle^41^. In a normal brain, both astrocytes and neurons respond to increased extracellular glutamate to transmit vasoactive signals to conduct vasodilation or vasoconstriction for the distribution of CBF^42,43^. On the other hand, a condition, such as a local functional ischemia is likely impaired due to dysregulation of glutamate transmission between the glutamatergic synapse and the metabotropic glutamate receptors (mGluRs) on astrocytes, resulting in abnormal blood flow after muscle contraction. Functional connectivity analysis of our hemodynamic data revealed abnormal blood metabolism and weakened connectivity strength in the PFC in chronic HD patients. This macroscopic interpretation may reflect neurovascular abnormality associated with functional ischemia in PFC in opiate-using patients. Indeed, our hemodynamic data are in line with prior neuroimaging studies^3,44–46^ consistently reporting that decreased rCBF in the frontal cortex was found in opiate-dependent subjects, and suggesting that opiate addiction was associated with increased risk of cerebrovascular ischemia. These pathological findings may be due to recurrent opiate-induced episodes of hypoxias^1,47,48^. Future studies with advanced neuroimaging techniques, such as resting-state magnetic resonance imaging (MRI)^12^, or single-photon emission computed tomography^49^, computed tomography angiography^50^, may enhance and confirm the diagnosis of functional ischemia in blood vessels of the cortex associated with opiate addiction.

Oscillatory desynchronization is induced by disorganizing information (neuronal or hormonal) flow, which is thought to result from dysfunction in the glutamine-GABA balance between brain regions^51^. We found global desynchronization of EEG networks in a large spectrum of low frequency bands comprising delta, theta, and lower alpha bands in HD patients. CBPT analysis also revealed local desynchronization in the lower alpha frequency band in the patient group. One reason could be that long-distanced interactions incline to involve synchrony in low frequencies^52,53^, putting less constraints on the timing precision due to long phases of enhanced and reduced excitability^54^. Although this mechanism is unknown, disturbed neural oscillations at these low frequencies are thought to relate to disable coordinated activity during normal functioning^55^.

Further, alpha rhythms, the most prominent over occipitoparietal cortex, especially during eye-closed and relaxed alert states^56^, is generated in thalamus^57,58^, in which its projection to the cortex drives and modulates cortical activity, such as control of cognition and social behavior^59^. This thalamocortical alpha synchrony results from reciprocal interactions between glutamatergic and GABAergic neurons whereby the synchronization is sustained by gap junctions among GABAergic interneurons^60,61^. This mechanism is modulated, in part, by glutamatergic afferents acting via mGluRs^61,62^. Thus, this explanation reconciles our quest that dysregulation of glutamatergic transmission may be, in large part, on astrocytes^63^, and that glutamine astrocytes activation may play an important role in long-term withdrawal in OUD^64,65^. It opens a door for future neuroplastic studies to navigate the astroglial proinflammatory response as well as the role of GABAergic interneurons in the corticolimbic circuitry^66^ by chronic opiate insult and its anti-inflammatory response by abstinence. Furthermore, disturbed alpha synchronization has also been showed in bipolar disorder^59^, attention deficit hyperactivity disorder^67^, schizophrenia^68^, Alzheimer’s disease^69^, and Fragile X syndrome^70^ (for a review in neural synchrony in brain disorders, see Uhlhaas *et al*.^71^). Hence, the heroin-induced local desynchronization in the lower alpha rhythm from our EEG data supports the notion that brain oscillations can serve as biomarkers in neuropsychiatric disorders, in our case opiate addiction. It also suggests that local synchronization in the upper alpha rhythm in PFC may contribute a sign of recovery after prolonged cessation from heroin. The EEG-fNIRS bimodality can be advantageous for brain state-dependent electrotherapy^72^, as well as predict responses to emotional cues^73^ and behavior performance^74^ in OUD rehabilitation. This computational approach is based on the assumption that homeostasis of the brain microenvironment is maintained through blood vessels, neurons, and astrocytes by the excitation-inhibition balance in the microcircuits. Notably, during abstinence, our fusion data, in line with previous cellular neuroadaptation literature in chronic opiate during tolerance and withdrawal suggesting that hemostatic adaptation in glutamate-GABA balance is developed through neural and neuron-glia network in opiate addiction^2^, may be a result of disturbed glutamate hemostasis, leading to the enhanced lower-alpha amplitude in the right FP and depressed in the left FP of PFC in HD patients. Further, in a given brain region, the power of alpha oscillations increases as the overall neuronal activity decreases^75^. Hence, the asymmetric excitability in PFC in HD patients revealed the profound effect of chronic heroin and recapitulated the glutamate hemostasis hypothesis. Some structural neuroimaging studies also showed asymmetry in PFC in abstinent heroin addicts in which reduced grey-matter density was found in the right PFC^76^. Although the study of relationship between structural and functional changes in brain network is still ongoing^77^, one multimodal neuroimaging study found that diminished gray-matter volume was positively associated with low perfusion in frontal area in heroin-dependent subjects^78^. Future studies in this direction will provide important insights to achieve structural and functional connectivity in large-scale networks^77^, as well as their relevance to cognition and behavior.

Although we provide a mesoscopic-scale functional PFC network in heroin addiction during protracted abstinence, neurovascular coupling in humans is still poorly understood^41^, and the use of rsFC in SUDs^11^ and the EEG-fNIRS machine learning approach^79^ are still at their infant stage. Findings presented in this work must be treated with caution until results are confirmed in a larger sample using advanced techniques. Though, fNIRS has some exclusive properties that may be leveraged over other neuroimaging techniques, such as fMRI, because fNIRS offers biochemically specificity by measuring concentrations of HbO and Hb and can be beneficial when localizing brain activity, especially in situations in which drug-dependent patients who may have difficulty to lie patiently in a magnet scanner. The optical method opens a door for clinical studies working with patients with attention deficits. Monitoring CBO over several hours or weeks in patients’ forehead at walkable pace perhaps is a prerequisite for the detection of glutamine balance and thus relapse in patients with drug addiction during stress attacks/exposure^80^ which may lead to the reinforcement of the negative-affective states^8^. Hence, the ability to detect glutamine balance in the PFC could eventually lead to an application for portable and cost-effective optical neuroimaging in clinical psychiatry to predict relapse on a possible recovering journey.

## Materials and Methods

### Study design

This study focuses on heroin-dependent patients (HD) who had exposed to heroin for over 20 years and who managed to stay abstinent for at least three months. They were compared with the healthy subjects in control group (CG) enrolled with demography matching the recruited HD patients with age, education level, and IQ (Fig. 1A).

This is an exploratory study to assess the resting-state neural synchrony, electrical oscillations, the cerebrovascular effect, and the functional connectivity of HD patients after protracted abstinence. This study is based on the assumption that neuronal activity is generated by electrical field and greatly relies on the neurovascular coupling mechanism. Hence, through modern machine learning method, the study is to design to examine neurophysiological information in the PFC in opiate addiction during abstinence through electrophysiological and neurovascular neuroimaging techniques. The work flows of EEG and fNIRS data processing analyses are showed in Fig. 1B.

### Study subjects

HD patients were eligible if they were diagnosed of OUD by a physician, had a history of at-least-20-years of recurrent heroin use, stayed abstinent for at least 3 months by the time of the study and were righted-handed between 39 and 57 years old. Control subjects were enrolled with demographic factors (i.e., age, education level, and IQ) matching the recruited HD patients (Fig. 1A). Prior to inclusion in the study, all enrolled subjects were required to complete a preclinical interview and collect a urine sample for urinalysis. The Structured Clinical Interview for DSM-IV (SCID) was administrated in the interview to verify opioid dependence and mental-state suitability for the study. Subjects with history of any neurological illness, head trauma, brain injury, or psychiatric disorders other than OUDs and Tabaco use disorder, and 72-hour of psychoactive substance use before any experimental measurements were excluded. The protocol was approved by the Medical Ethics Committee of the University of Macau. The experiments were performed in accordance with the relevant guidelines and regulations in the latest version of the Declaration of Helsinki. Written informed consents were provide to all subjects. Subjects were introduced and instructed to sit comfortably on a chair in a quiet and dim room located at the Bioimaging Core of the Faculty of Health Sciences where the study was conducted. EEG and fNIRS signals in the eye-closed resting state were simultaneously and continuously recorded for 11 minutes. The first 2-minute measurements^81,82^ were eliminated to generate relatively steady signals and rule out potential effects of instability for analysis. In part of Fig. 1B shows the configuration of the EEG electrodes and fNIRS channels. A three-dimensional (3-D) digitizer (Polhemus Inc., Vermont) was used to complete the spatial registration of the electrode and channel locations, and thus, the MNI coordinates were obtained (see Supplementary Table S1).

### Electrophysiological measurements

The electrical activity of manifold neurons up to large neuronal assemblies can be directly measured non-invasively with extracellular electrophysiological recordings, such as MEG^83^ or EEG^59^, which are primarily generated by post-synaptic potentials and thus often susceptible to changes in neurotransmission secondary to neural dysfunction or neuropharmacological manipulations. The EEG recordings were conducted on the Biosemi ActiveTwo system (Biosemi, Amsterdam, The Netherlands; sampling rate: 2048 Hz) from 64 electrodes on the International 10–20 system with a nose reference based. The horizontal and vertical electrooculogram (EOGs) were additionally acquired to oversee eye movements. Electrode impedance was less than 10 kΩ.

EEG data were preprocessed using EEGLAB (https://sccn.ucsd.edu/eeglab). Ocular artifacts acquired by EOGs channels were corrected in the recorded EEG data^84^. The EEG data were segmented into 2-second epochs, of which with voltage exceeded ± 150 µV were excluded^59^. Frequency bands of interest were filtered and classified by delta (1.5–4 Hz), theta (4–8 Hz), lower alpha (8–10 Hz), upper alpha (10–13 Hz), beta (14–30 Hz), and gamma (30–50 Hz). Classification of alpha oscillations in lower and upper alpha frequency bands offers a narrowed range of interest. EEG data were then down-sampled from 2048 to 256 Hz. For each subject, the first 10 artifact-free baseline-corrected epochs (5120 samples; 20 seconds) were selected for further processing and analysis. The detailed preprocessing method is documented in Kim *et al*.^59^.

Because neuronal oscillations have been associated practically with many aspects of cognitive function^56,85,86^, two important and distinct temporal variables from the EEG: neural synchrony and oscillations were the subjects of investigation in the study.

### Hemodynamic measurements

Neurovascular activity, often known as hemodynamic activity, can be indirectly measured without exogenous contrast agents non-invasively by functional MRI^87^ or functional intrinsic optional imaging, such as fNIRS^22^. The fNIRS recordings were conducted on the NIRS Continuous Wave (CW) system (CW6 fNIRS system; TechEn Inc, Milford, MA; sampling rate: 50 Hz; bandpass range: 0.1–200 Hz), with four sources and eight detectors to generate fourteen channels on the scalp, covering the PFC. Each set of a source and detectors covered one particular PFC subregions, resulting in four ROIs: the right dlPFC, the left FP, the right FP, and the left dlPFC (see Supplementary Table S1 for details). The source-detector distance was 3 cm, measuring the neural activity of the cortex with an estimated 2.5 cm penetration depth^88^. The fNIRS monitors hemodynamic responses evoked by brain activity by using two CW lights at wavelengths of 690 nm and 830 nm - the characteristic absorption patterns in the near-infrared light, to obtain quantitative data of HbO and Hb.

fNIRS data were preprocessed using Homer2 (www.homer-fnirs.org/documentation). The preprocessed procedures were as follow: (1) Conversion from optical density to the changes in HbO or Hb concentration at different time points based on the modified Beer-Lambert Law^89^; (2) Bandpass filtering (0.01 < fr < 0.1 Hz); (3) Detrending; and (4) Motion correction using the spline interpolation method. There are two main types of noise due to poor contact between the optodes and scalp: motion artifacts and a low signal-to-noise ratio (SNR). And thus, quality control is necessary before functional connectivity analysis. To reduce the motion-induced artifacts, the spline interpolation method detected the artifacts by calculating the moving standard deviation within sliding time windows in a 2-seoncd window length. Any data with average signal intensity over five standard deviations of the HbO and Hb concentrations over time are considered low SNRs and thus should be excluded. The detailed preprocessing method is documented in Niu *et al*.^90^. No channel was excluded due to poor contact. Changes in HbO and Hb concentrations were extracted from each channel across all subjects to visually verify if there were changes of these molecules to reflect brain functional activity (i.e., an increase in [HbO] and a decrease in [Hb] indicate typical CBO, and thus brain activation).

### Functional connectivity analyses

Functional connectivity is a statistical dependence between the time series of measured neurophysiological signals^91,92^. Two points in a brain are considered functionally connected if they have synchronized or coherent dynamics. The translation of different measures in the neurophysiological signals depends on the modality of recordings being used. In this study, we used the measure of SL in electrophysiological signal on the EEG recording and the measures of change in both HbO and Hb concentrations in neurovascular signal on the fNIRS recording.

To analyze the rsFC electrophysiological signal, the temporal information from the EEG, such as synchrony, was measured by SL. It is a generalized synchronization approach that can detect linear and nonlinear interdependences between two signals in a given number of electrodes^23^, especially sensitive to the long-distanced synchronization of structured oscillatory activities of across whole-brain areas. SL in each frequency band as a measure of rsFC across time and electrode and then the functional connectivity were computed using the HERMES toolbox (http://hermes.ctb.upm.es/). SL values of each subject were then averaged across each pair nodes. Permutation test was performed to determine the between-group difference at rest. Mean SL was analyzed to determine which frequency band was significant between groups, and its band power would be selected for further machine learning method. Connection-wise SL was analyzed by performing CBPT for multiple comparisons not only to identify the between-group significance where the SL value in HD group was significant compared to the controls, but also to offer a powerful and intuitive nonparametric framework for statistical analysis of power spectra in what brain region. Methodological details can be found in Nico *et al*.^93^. The electrodes corresponding to the significant brain region (i.e., source) calculated by CBPT would be selected for machine learning method.

To analyze the rsFC neurovascular effect, the information regarding to the changes in HbO and Hb concentrations from the fNIRS were evaluated. Whole-brain correlation analysis was performed because it allowed us to easily observe the change of the two chromophores in a matrix and, more importantly, to compute a functional connectivity map between any two channel pairs in the PFC system, where the channels represented nodes, and where the functional connections with correlation coefficient, *r*, greater than a predefined threshold were considered edges. The result of computing *r* for each channel of a chromophore was a 14 × 14 matrix. All correlation values were converted to z-scores using the Fisher z-transformation^94^ prior to any statistical analysis. For simplicity, the concentration change in HbO was primarily analyzed and selected for further processing in the subsequent quantitative nodal network analysis as a feature for machine learning method because it is thought to be the most responsive indicator of differences in CBF and the most sensitive parameter of change in rCBO^95^. Consisting with our previous work, the threshold value was determined by the foremost 5% of the connections before generating a network topological map. For visualization, BrainNet Viewer toolbox (https://www.nitrc.org/projects/bnv) was applied. For nodal network analysis, the degree strength of each channel was quantified by averaging connectivity of a channel in the PFC system. Methodological details can be found in our previous work^24,96^.

### Machine learning: multi-modal data analysis for PFC imaging

One of the most modern multimodal neuroimaging setups is to combine the measurement of electrophysiology with the measurement of hemodynamics^97^. Machine learning method is used to obtain correlations between EEG and fNIRS modalities by extracting physiologically plausible components^25^. It is believed that the data obtained through this multimodality which otherwise offers exceptional spatial and temporal imaging resolution allows for combination of complementary information, thereby a desirable diagnosis, and a deeper understanding of how electrophysiological and neurovascular aspects of brain activity are related.

To increase precision and detect minuscule signals from both of the electrophysiological and hemodynamic measurements, the mSPoC analysis^25^ was performed. The amplitude (i.e., band power) and the frequency of the temporal oscillation selected from the EEG-based connectivity analysis, were extracted as an EEG feature. In order to perform the bi-modal EEG-fNIRS fusion, the number of samples from fNIRS data must equal to the number of epoch from EEG data because the modulations of a source extracted from fNIRS is related to the modulations of the power of a source from EEG. The preprocessed fNIRS data were then down-sampled from 50 to 5 Hz. For each subject, the first 10 HbO-based samples (i.e., [HbO_f_]) were extracted as a fNIRS feature (in part, Fig. 1B). Both datasets were preprocessed to extract features using a sliding window of 2 seconds. The mSPoC method was proposed to examine the comodulation between resting-state-induced power dynamics of the selected oscillatory rhythm and changes in HbO concentration. Through this supervised machine learning strategy, it finds the direction (i.e., spatial filters) of the two class (i.e., EEG and fNIRS) in a high-dimensional space such that the spectral power dynamics of the projected electrophysiological signal (i.e., EEG-source) maximizing covaried with the time-course of the project hemodynamic signal (i.e., a corresponding fNIRS-source). The time-invariant coupling coefficients of each source with the different sensors (i.e., the common spatial patterns) of EEG and fNIRS data were then calculated. The detailed fusion method is documented in Dähne *et al*.^25,97,98^. The brain area generating the EEG mSPoC component was localized in the PFC subregions and across PFC region as a whole. The EEG mSPoC component which has the greatest coupling coefficient value was then selected to fuse with the corresponding fNIRS component. In addition to mSPoC analysis, sensor-space cortical source estimation was separately performed for both EEG and fNIRS datasets by using the eConnectome toolbox (http://econnectome.umn.edu/) and the NIRS-SPM toolbox (http://bispl.weebly.com/nirs-spm.html#/), respectively, to visually scan through activation locations. The activation pattern of the fused EEG and fNIRS component estimated by mSPoC method was then visualized by using BrainNet Viewer (https://www.nitrc.org/projects/bnv).

### Statistical analysis

Statistical analyses were performed using SPSS (IBM SPSS Statistics, version 22, Armonk, NY). Group differences in demographic and ROI data were investigated using two-sample *t* test and Bonferroni correction. Statistical permutation test was performed throughout the study for connectivity analysis and machine learning method to determine group difference between two means in which the null distribution of test statistic was obtained by a number of random rearrangements for each study group (i.e., permutations)^59,93^. It worked as follows. First, a *t*-test was performed at *p* < 0.05 significance level (uncorrected for multiplicity) to give an initial approximation to the study of statistical differences between the HD and control groups. Second, each subject was randomly reassigned to either a patient or a control group, thereby forming a division that was no longer faithful to the original real partition of the data sets. The procedure was repeated 1,000 times. The *t*-values were recalculated, and the null distribution of test statistics for the group difference was obtained. Finally, the proportion of sampled permutations where the *t*-values were larger than the observed test statistics was computed as the *p*-value of the observed group difference. Similarly in mSPoC analysis, permutation was performed to randomize the initial set of EEG-signal spatial filters until a suitable convergence criterion was met in order to optimize the fusion objective^25^. A level of significance was set *p* < 0.05 (uncorrected), and subject’s age, gender, IQ were defined as covariates to minimize potential biases to the results. Due to the small sample size of the study, we used the current alternative strategy for convention^99,100^, in which the matched sample size was appropriately adapted to the cost concerns of the study. All *p* values were two-tailed (α = 0.05). MATLAB (MathWorks, Inc., Natick, MA, USA) was used for all computations.

## Supplementary information

Supplementary Information

Supplementary Information
