# Supplementary material for: Machine learning: assessing neurovascular signals in the prefrontal cortex with non-invasive bimodal electro-optical neuroimaging in opiate addiction
Source: Sci Rep. 2019 Dec 4;9:18262. doi: 10.1038/s41598-019-54316-6 (PMC6892956; doi:10.1038/s41598-019-54316-6)

Machine learning: assessing neurovascular signals in the prefrontal cortex with non-invasive bimodal electro-optical neuroimaging in opiate addiction

Hada Fong-ha Ieong[1†*], Fu Gao[2], Zhen Yuan[1,3*]

[1]Bioimging Core, Faculty of Health Sciences, University of Macau, Taipa, Macau S.A.R.

[2]Department of Cardiac Surgery, Yale School of Medicine, Yale University, New Haven, CT, U.S.A.

[3]Centre for Cognitive and Brain Sciences, Institute of Collaborative Innovation, University of Macau, Taipa, Macau S.A.R.

[†]Present Address: Department of Anesthesiology, Yale School of Medicine, Yale University, New Haven, CT, U.S.A.

*Correspondence: hada.ieong@connect.um.edu.mo ; zhenyuan@um.edu.mo

Contents

Supplementary Table S1 **2**

Supplementary Table S2 **3**

Supplementary Table S3 **4**

Supplementary Figure S1 **5**

Table S1. Summary of the four Region of Interest (ROI) and their associated fNIRS channels and EEG electrodes and their MNI coordinates.

<table>
<tr><th>ROIs</th><th>fNIRS Channel</th><th colspan="3">Channel Coordinates (MNI)</th><th>fNIRS Automated anatomical labeling</th><th>EEG electrode</th><th colspan="3">Electrode Coordinates (MNI)</th><th>EEG Automated anatomical labeling</th><th>BA</th></tr>
<tr><td rowspan="4">Left dlPFC</td><td>1</td><td>35</td><td>62</td><td>15</td><td>Left middle frontal gyrus</td><td>AF7</td><td>-44</td><td>57</td><td>-3</td><td>Left middle frontal gyrus, orbital part</td><td>46</td></tr>
<tr><td>2</td><td>-46</td><td>42</td><td>29</td><td>Left middle frontal gyrus</td><td>F5</td><td>-52</td><td>39</td><td>19</td><td>Left inferior frontal gyrus, triangular part</td><td>46/45</td></tr>
<tr><td>3</td><td>-33</td><td>54</td><td>31</td><td>Left middle frontal gyrus</td><td>F3</td><td>-40</td><td>42</td><td>37</td><td>Left middle frontal gyrus</td><td>46</td></tr>
<tr><td colspan="5">-</td><td>AF3</td><td>-26</td><td>64</td><td>23</td><td>Left superior frontal gyrus</td><td>46</td></tr>
<tr><td rowspan="4">Left FP</td><td>4</td><td>-21</td><td>69</td><td>17</td><td>Left superior frontal gyrus</td><td>FP1</td><td>-23</td><td>70</td><td>-1</td><td>Left superior frontal gyrus</td><td>10</td></tr>
<tr><td>5</td><td>-9</td><td>72</td><td>16</td><td>Left superior medial frontal gyrus</td><td>AFz</td><td>2</td><td>64</td><td>27</td><td>Left superior frontal gyrus</td><td>10</td></tr>
<tr><td>6</td><td>-19</td><td>61</td><td>33</td><td>Left superior frontal gyrus</td><td>F1</td><td>-18</td><td>45</td><td>50</td><td>Left superior frontal gyrus</td><td>10</td></tr>
<tr><td>7</td><td>-7</td><td>63</td><td>35</td><td>Left superior medial frontal gyrus</td><td colspan="5">-</td><td>10</td></tr>
<tr><td rowspan="4">Right FP</td><td>8</td><td>11</td><td>72</td><td>17</td><td>Right superior frontal gyrus</td><td>FPz</td><td>1</td><td>69</td><td>1</td><td>Right superior frontal gyrus</td><td>10</td></tr>
<tr><td>9</td><td>25</td><td>70</td><td>16</td><td>Right superior medial frontal gyrus</td><td>FP2</td><td>26</td><td>70</td><td>-2</td><td>Right superior frontal gyrus</td><td>10</td></tr>
<tr><td>10</td><td>11</td><td>63</td><td>35</td><td>Right superior medial frontal gyrus</td><td>Fz</td><td>2</td><td>47</td><td>50</td><td>Right medial frontal gyrus</td><td>10</td></tr>
<tr><td>11</td><td>23</td><td>60</td><td>33</td><td>Right superior frontal gyrus</td><td>F2</td><td>22</td><td>45</td><td>51</td><td>Right superior frontal gyrus</td><td>10</td></tr>
<tr><td rowspan="4">Right dlPFC</td><td>12</td><td>41</td><td>60</td><td>15</td><td>Right middle frontal gyrus</td><td>AF8</td><td>48</td><td>55</td><td>-2</td><td>Right middle frontal gyrus, orbital part</td><td>46</td></tr>
<tr><td>13</td><td>39</td><td>51</td><td>32</td><td>Right middle frontal gyrus</td><td>AF4</td><td>31</td><td>61</td><td>26</td><td>Right middle frontal gyrus</td><td>46</td></tr>
<tr><td>14</td><td>51</td><td>40</td><td>27</td><td>Right middle frontal gyrus</td><td>F6</td><td>55</td><td>36</td><td>21</td><td>Right inferior frontal gyrus, triangular part</td><td>46/44</td></tr>
<tr><td colspan="5">-</td><td>F4</td><td>44</td><td>36</td><td>40</td><td>Right middle frontal gyrus</td><td>46</td></tr>
</table>

Channel coordinates were generated using the NIRS-SPM software (http://bispl.weebly.com/nirs-spm.html#/) after registration using a 3D digitizer (Polhemus Inc., VT). Automated anatomical labeling (AAL) is applied.

Table S2. Group-Differences in nodal degree strength in the change of HbO Concentration (ROI analysis) during resting state.

ROIs	fNIRS Channel	fNIRS Automated anatomical labeling	CG (n = 11)	HD (n = 8)	*P* *Two-tailed*
**Left dlPFC**	**1**	Left middle frontal gyrus	0.491 (0.056)	0.280 (0.054)	0.018*
	**2**	Left middle frontal gyrus	0.642 (0.046)	0.491 (0.080)	0.099
	**3**	Left middle frontal gyrus	0.749 (0.040)	0.494 (0.078)	0.006*
**Left FP**	**4**	Left superior frontal gyrus	0.478 (0.043)	0.254 (0.051)	0.004*
	**5**	Left superior medial frontal gyrus	0.732 (0.035)	0.234 (0.064)	0.0001**
	**6**	Left superior frontal gyrus	0.658 (0.045)	0.479 (0.087)	0.065
	**7**	Left superior medial frontal gyrus	0.684 (0.040)	0.524 (0.077)	0.063
**Right FP**	**8**	Right superior frontal gyrus	0.813 (0.034)	0.085 (0.070)	0.0001**
	**9**	Right superior medial frontal gyrus	0.871 (0.040)	0.610 (0.073)	0.004*
	**10**	Right superior medial frontal gyrus	0.807 (0.037)	0.561 (0.070)	0.004*
	**11**	Right superior frontal gyrus	0.835 (0.046)	0.608 (0.068)	0.011*
**Right dlPFC**	**12**	Right middle frontal gyrus	0.738 (0.043)	0.538 (0.072)	0.022*
	**13**	Right middle frontal gyrus	0.686 (0.052)	0.483 (0.072)	0.031*
	**14**	Right middle frontal gyrus	0.621 (0.046)	0.335 (0.070)	0.002*

The p values are reported for a two-sample t test (for age, years of education and IQ; two-tailed) comparing abstinent heroin-dependent subjects (HDs) with the controls (CGs). Mean and standard error of the mean (s.e.m.) are reported.

Table S3. Group-Differences in nodal degree strength in the change of Hb Concentration (ROI analysis) during resting state.

ROIs	fNIRS Channel	fNIRS Automated anatomical labeling	CG (n = 11)	HD (n = 8)	*p*
**Left dlPFC**	**1**	Left middle frontal gyrus	0.292 (0.057)	0.114 (0.051)	0.040*
	**2**	Left middle frontal gyrus	0.341 (0.036)	0.448 (0.061)	0.127
	**3**	Left middle frontal gyrus	0.379 (0.048)	0.436 (0.059)	0.460
**Left FP**	**4**	Left superior frontal gyrus	0.289 (0.063)	0.253 (0.071)	0.711
	**5**	Left superior medial frontal gyrus	0.251 (0.056)	0.209 (0.057)	0.614
	**6**	Left superior frontal gyrus	0.314 (0.057)	0.500 (0.063)	0.045*
	**7**	Left superior medial frontal gyrus	0.248 (0.055)	0.266 (0.077)	0.847
**Right FP**	**8**	Right superior frontal gyrus	0.415 (0.039)	0.168 (0.046)	0.0007**
	**9**	Right superior medial frontal gyrus	0.407 (0.056)	0.452 (0.059)	0.534
	**10**	Right superior medial frontal gyrus	0.299 (0.041)	0.382 (0.064)	0.268
	**11**	Right superior frontal gyrus	0.402 (0.055)	0.368 (0.066)	0.696
**Right dlPFC**	**12**	Right middle frontal gyrus	0.317 (0.058)	0.424 (0.073)	0.262
	**13**	Right middle frontal gyrus	0.298 (0.049)	0.451 (0.067)	0.076
	**14**	Right middle frontal gyrus	0.152 (0.048)	0.222 (0.076)	0.425

The *p* values are reported for a two-sample *t* test (for age, years of education and IQ; two-tailed) comparing abstinent heroin-dependent subjects (HDs) with the controls (CGs). Mean and standard error of the mean (s.e.m.) are reported.

Figure S1. Oxygen metabolism comparison by channel in HD patients and the controls.

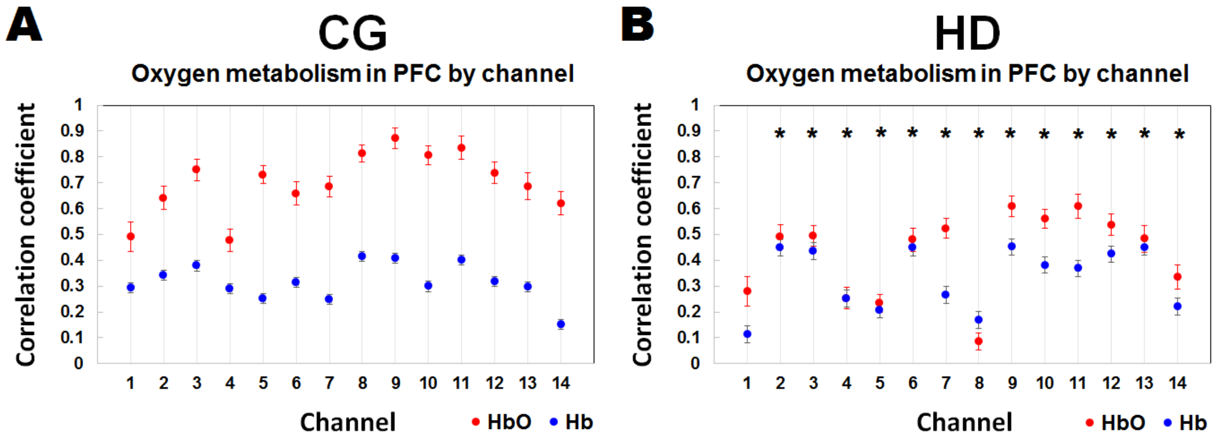

Supplement: Supplementary file 1 — Supplementary Information [file 41598_2019_54316_MOESM1_ESM.pdf]
